# Awareness and associated factors of venous thromboembolism in breast cancer surgical patients: a cross-sectional study

**DOI:** 10.1186/s12885-024-12231-8

**Published:** 2024-05-21

**Authors:** Qiuzhou Wang, Hongxiu Chen, Qingyu Yang, Jiajia Qiu, Sijin Guo, Yi Zhou, Lihong Huang, Chen Li, Xiaoxia Zhang

**Affiliations:** 1 Division of Breast Surgery, Department of General Surgery, West China Hospital, Sichuan University, Chengdu, China; 2 Breast Center, West China Hospital, Sichuan University, Chengdu, China; 3 Innovation Center of Nursing Research, Nursing Key Laboratory of Sichuan Province, West China Hospital, Sichuan University, Chengdu, China; 4 West China School of Nursing, Sichuan University, Chengdu, China; 5 Department of Nursing Administration, Shanghai Cancer Center, Fudan University, Shanghai, China; 6 Department of Thyroid, Breast and Vascular Surgery, Xijing Hospital, Air Force Medical University, Xi’an, China; 7 Department of Breast Surgery ward II, Affiliated Hospital of Guizhou Medical University, Guiyang, China; 8 Division of Breast Surgery, Department of General Surgery, PLA General Hospital, Beijing, China; 9 Department of Gynecology, the First Affiliated Hospital of Kunming Medical University, Kunming, China; 10 Division of Head & Neck Tumor Multimodality Treatment, Cancer Center, West China Hospital, Sichuan University, Chengdu, China

**Keywords:** Breast cancer, Venous thromboembolism, Deep vein thrombosis, Pulmonary embolism, Awareness level

## Abstract

**Background:**

Venous thromboembolism (VTE) is a major complication of breast cancer surgical patients. Assessing VTE awareness enables medical staff to tailor educational programs that improve patient self-management and reduce VTE risk. Therefore, this study aimed to assess VTE awareness among breast cancer surgical patients and identify factors influencing their awareness level.

**Methods:**

A multicenter cross-sectional study was conducted on breast cancer patients scheduled for surgery from May 2023 to November 2023. Data were collected using a general information form and a validated self-assessment questionnaire on VTE awareness for breast cancer surgical patients. Univariate analysis and multiple linear regression analysis were used to analyze the data.

**Results:**

Of 1969 patients included, the term awareness rates for deep vein thrombosis and pulmonary embolism were 42.5% and 26.1%, respectively. Information about VTE was primarily obtained from doctors (30.4%), nurses (24.0%), and social media (23.3%). The overall average VTE awareness score was 1.55 ± 0.53, with the dimension of VTE preventive measures scoring highest, and VTE clinical symptoms/signs scoring lowest. Multivariate analysis identified education level, personal VTE history, chemotherapy and surgical history, and the hospital’s regional location as significant factors associated with VTE awareness level (*p* < 0.05).

**Conclusion:**

This study highlights a critical need for improved VTE awareness among breast cancer surgical patients, particularly regarding clinical symptoms/signs. Health education programs are recommended especially tailored for patients with lower education levels, no history of VTE, or without prior surgery or chemotherapy, to improve their understanding of VTE.

## Background

Venous thromboembolism (VTE), encompassing deep vein thrombosis (DVT) and pulmonary embolism (PE), significantly contributes to the global disease burden [1]. VTE related to hospitalization is a major cause of mortality and morbidity across both developed and developing nations [2]. Given its high morbidity, mortality, and associated healthcare costs, numerous international, national, and regional organizations have implemented various measures, such as setting up *World Thrombosis Day* by the International Society on Thrombosis and Haemostasis, publishing the *Call to Action to Prevent DVT and PE* by the United States Surgeon General, and established the *National Program for Prevention and Management of PE and DVT* by Chinese government, to raise the awareness of thrombosis disease among medical professions and the public [3–5].

Breast cancer, which has the highest prevalence worldwide, also sees a significant number of patients developing VTE, accounting for 17% of all cancer patients receiving anticoagulation therapy [6]. Previous studies have indicated that VTE could disrupt the course of anticancer treatment, extend the hospitalization days by about a week, increase by 2919 ~ 3611€ treatment cost per patient, and even increase the risk of death of breast cancer patients [7–11]. Surgery, a principal treatment modality for breast cancer, is also widely acknowledged as a significant risk factor for VTE. The incidence of VTE in breast cancer patients after surgery has been reported as high as 31.4% [12], with the majority of cases occurring within one month postoperatively [13–15]. Therefore, breast cancer patients undergoing surgery constitute a key group for the prevention and control of VTE.

Although the clinical features [16], risk factors [17–21], and prophylactic measures [22–26] of VTE in breast cancer patients undergoing surgery have been studied, no research was found which reported the awareness level of VTE and the knowledge level related to VTE clinical manifestation, key risk factors, prophylactic measures, etc. in patients with breast cancer. Assessing patients’ awareness of VTE can assist healthcare providers in developing more targeted and precise health education programs, to attract patients to actively participate in VTE prevention. Consequently, this study aims to (1) investigate the awareness level of VTE among breast cancer patients undergoing surgery, and (2) identify factors associated with their level of awareness.

## Methods

### Study design and setting

This was a multi-center cross-sectional study conducted in 20 provinces, involving 47 hospitals. This study received approval from the Ethics Committee of West China Hospital,Sichuan University (Approval No. 2023 − 1276) and adhered to the Strengthening the Reporting of Observational Studies in Epidemiology (STROBE) guidelines.

### Participants

Inpatients with breast cancer planning to receive a mastectomy from May 2023 to November 2023 were eligible for this survey. The inclusion criteria were as follows: 1) a pathological diagnosis of breast cancer; (2) age ≥ 18 years; 3) consciousness and ability to complete the questionnaire independently or with investigator assistance; and 4) voluntary participation in the survey. Patients who could not fill out the questionnaire with the assistance of the investigator were excluded. All participants signed the electronic or paper informed consent form.

### Sample size calculation

The sample size of this study was calculated based on the calculation method used for the cross-sectional study, i.e. $N=\frac{{Z}_{1-\alpha /2}^{2}\left(1-p\right)}{{\epsilon }^{2}p}$. Considering that there is no research reported on the awareness rate of VTE in breast cancer patients, we referred to the results of a survey conducted on cancer patients in internal medicine, which reported the awareness rate of DVT was 28.8% [27]. Therefore, we took 28% as the value of proportion (*p)* in this study. Additionally, the value of confidence level (1-α) and admissible error (δ) were taken to the generally accepted value of 95% and 10%, respectively. In summary, at least 988 patients were needed$(N=\frac{{1.96}^{2}\times \left(1-0.28\right)}{{0.1}^{2}\times 0.28}=988$).

### Measurements

#### General information form

A general information form was pre-designed to collect patients’ sociodemographic information (i.e. age, education level, and marital status), medical history (including personal VTE history, family history of VTE, surgical history, chemotherapy history, and central venous catheterization history), whether heard of the term or diseases of hypertension, hyperlipidemia, stroke, coronary heart disease, DVT, PE, and ways to learn about VTE.

#### VTE awareness level self-evaluation questionnaire

Based on the questionnaire developed by Wendelboe [28] and Aggarwal [29], a VTE awareness level self-evaluation questionnaire specialized for breast cancer patients was designed after a comprehensive literature review. Five clinical medical and nursing experts from the vascular or oncology department were invited to modify and test the content validity of this questionnaire. Then, a pilot survey was conducted on 40 breast cancer patients to check whether the descriptions of items were unclear or ambiguous. Finally, the questionnaire contained four dimensions, i.e., basic knowledge (13 items), clinical symptoms/signs (11 items), risk factors (17 items), and preventive measures of VTE (6 items), with a total of 47 items. Each item was rated on a 3-point Likert scale, where 1 point indicated ‘completely unknown’ and 3 points indicated ‘very known’. To compare these four dimensions easily, the scores of each dimension were standardized, that is, the score of each dimension was equal to the total score of each dimension divided by the number of items in each dimension. The higher the score, the higher the awareness level of patients. The scale of content validity of this questionnaire was 0.97. The Cronbach’s α coefficient for the total questionnaire was 0.97, and for four dimensions were 0.90, 0.95, 0.95, and 0.87, respectively.

### Data collection

Data were collected by an investigator using an electronic questionnaire on the day of admission. After the patient submitted the questionnaire, the investigator checked the completeness of the questionnaire on the same day and asked the patient to fill in the missing items in time to ensure the integrity of the data. An invalid questionnaire is defined as an answer with obvious logical errors or inconsistent answers. A total of 2005 patients were investigated in this study, of which 36 questionnaires were eliminated because of inconsistent answers, and 1969 valid questionnaires were finally recovered, with a recovery rate of 98.2%.

### Statistical analysis

Data analysis was performed using SPSS (version 26.0). Participants’ characteristics were presented as frequencies and proportions, and the awareness level of VTE of participants was presented as x ± SD. Univariate analyses (t-test or ANOVA) and multiple linear stepwise regression analyses were used to analyze the factors influencing participants’ awareness level of VTE. Variables with a p-value < 0.05 from the univariate analysis were included in the multivariate analysis. Two side *p* < 0.05 was considered statistically significant.

## Results

### Characteristics of participants

The analysis included 1969 hospitalized breast cancer surgery patients with a mean age of 49.7 ± 11.5 years; 83.7% were under the age of 60. The majority of participants had an educational level of junior/senior high school or above; 4.2% of participants had a history of VTE and 26.5% had a history of surgery. Detailed demographic information is presented in Table 1.

**Fig. 1:**
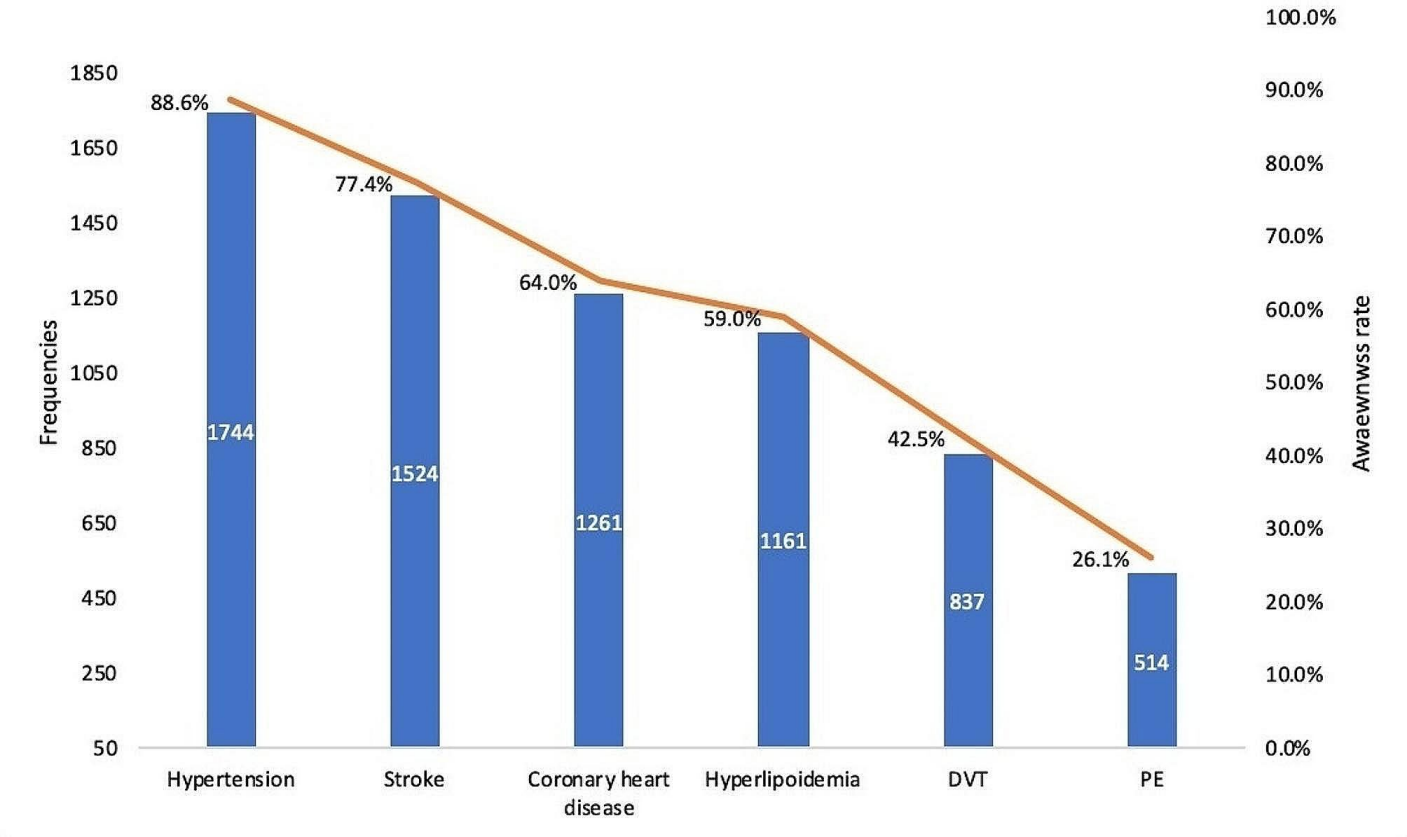
Terminology awareness related the cardiovascular diseases

Among the participants, a majority were familiar with the terms ‘hypertension’ (88.6%), ‘stroke’ (77.4%), and ‘coronary heart disease’ (64.0%). However, only 42.5% and 26.1% of them have heard the terms DVT and PE respectively (Fig. 1). Participants learned about VTE mainly through surgeons (*n* = 598), followed by nurses (*n* = 473), online social applications (*n* = 459), books or health brochures (*n* = 362), fellow patients or friends (*n* = 231), and other ways (*n* = 125).

**Table 1:** The VTE awareness level of participants with different characteristics and the univariate analysis results for the VTE awareness level

Variables [(n,%) or (x ± SD)]	Basic knowledge	Clinical symptoms/signs	Risk factors	Preventive measures	Total score
Age (years)					
< 60 (1648, 83.7%)	1.59 ± 0.53	1.49 ± 0.61	1.51 ± 0.56	1.78 ± 0.67	1.57 ± 0.54
≥60 (321, 16.3%)	1.53 ± 0.50	1.40 ± 0.55	1.42 ± 0.51	1.67 ± 0.62	1.48 ± 0.49
*P* value	0.047	0.011	0.007	0.005	0.008
**Education level**					
Primary school or beblow (249,12.6%)	1.35 ± 0.46	1.26 ± 0.47	1.31 ± 0.48	1.44 ± 0.60	1.33 ± 0.45
Junior/senior high school (837,42.5%)	1.49 ± 0.46	1.40 ± 0.53	1.41 ± 0.48	1.65 ± 0.61	1.46 ± 0.47
College graduated or above (883, 44.8%)	1.73 ± 0.56	1.62 ± 0.66	1.64 ± 0.60	1.96 ± 0.66	1.70 ± 0.57
*P* value	< 0.001	< 0.001	< 0.001	< 0.001	< 0.001
**Martial status**					
single (86, 4.4%)	1.96 ± 0.64	1.87 ± 0.77	1.90 ± 0.69	2.12 ± 0.72	1.94 ± 0.67
married (1764, 89.6%)	1.56 ± 0.52	1.46 ± 0.58	1.48 ± 0.54	1.74 ± 0.65	1.53 ± 0.52
other (119, 6.0%)	1.55 ± 0.50	1.49 ± 0.59	1.53 ± 0.57	1.82 ± 0.65	1.57 ± 0.53
*P* value	0.002	< 0.001	< 0.001	0.217	< 0.001
**Personal history of VTE**					
No (1887, 95.8%)	1.57 ± 0.52	1.46 ± 0.59	1.49 ± 0.54	1.75 ± 0.66	1.54 ± 0.53
Yes (82, 4.2%)	1.92 ± 0.59	1.83 ± 0.71	1.78 ± 0.66	2.02 ± 0.64	1.86 ± 0.60
*P* value	< 0.001	< 0.001	< 0.001	< 0.001	< 0.001
**Family history of VTE**					
No (1821, 92.5%)	1.56 ± 0.52	1.46 ± 0.59	1.48 ± 0.55	1.74 ± 0.65	1.53 ± 0.53
Yes (148, 7.5%)	1.84 ± 0.54	1.69 ± 0.67	1.72 ± 0.57	2.06 ± 0.68	1.79 ± 0.55
*P* value	< 0.001	< 0.001	< 0.001	< 0.001	< 0.001
**Surgical history**					
No (1448, 73.5%)	1.54 ± 0.52	1.43 ± 0.58	1.45 ± 0.54	1.71 ± 0.65	1.50 ± 0.52
Yes (521, 26.5%)	1.69 ± 0.53	1.60 ± 0.63	1.65 ± 0.56	1.93 ± 0.67	1.69 ± 0.54
*P* value	< 0.001	< 0.001	< 0.001	< 0.001	< 0.001
**Chemotherapy history**					
No (1233, 62.6%)	1.50 ± 0.50	1.41 ± 0.56	1.42 ± 0.52	1.68 ± 0.63	1.47 ± 0.50
Yes (736, 37.4%)	1.71 ± 0.54	1.59 ± 0.64	1.63 ± 0.58	1.91 ± 0.68	1.68 ± 0.56
*P* value	< 0.001	< 0.001	< 0.001	< 0.001	< 0.001
**CVC history**					
No (1505, 76.4%)	1.54 ± 0.52	1.44 ± 0.59	1.46 ± 0.54	1.72 ± 0.65	1.51 ± 0.52
Yes (464, 23.6%)	1.72 ± 0.53	1.60 ± 0.63	1.64 ± 0.56	1.92 ± 0.66	1.69 ± 0.54
*P* value	< 0.001	< 0.001	< 0.001	< 0.001	< 0.001
**Location**					
Eastern China(209, 15.7%)	1.82 ± 0.49	1.63 ± 0.63	1.73 ± 0.54	2.06 ± 0.63	1.78 ± 0.51
Central China(70, 3.6%)	1.90 ± 0.54	1.81 ± 0.74a	1.85 ± 0.62	2.12 ± 0.69	1.89 ± 0.58
Western China(1590, 80.8%)	1.52 ± 0.52	1.43 ± 0.57	1.44 ± 0.53	1.69 ± 0.64	1.49 ± 0.52
*P* value	0.218	< 0.001	< 0.001	0.009	< 0.001

VTE venous thromboembolism; CVC central venous catheterization

### The VTE awareness level of participants

Table 1  summarizes the total score and scores for 4-dimension scores of VTE awareness level under different characteristics of participants. The total score of all participants after standardization was 1.55 ± 0.53. The scores of four dimensions from high to low were: preventive measures of VTE (1.76 ± 0.66), basic knowledge (1.58 ± 0.53), risk factors (1.50 ± 0.55), and clinical symptoms/signs (1.48 ± 0.60). Table 2 presents the items with the highest and lowest scores within each dimension.

**Table 2:** Details of the scores of each item in each dimension

The three highest-scoring items in each dimension	Scores
Dimension of basic knowledge	
Do you know that a thrombus is an abnormal clotting of blood within the vessel?	1.79
Do you know that VTE can be treated?	1.74
Do you know that VTE can be prevented?	1.73
**Dimension of clinical symptoms/signs**	
Do you know that pulmonary embolism may present with dyspnea or shortness of breath?	1.56
Do you know that DVT may present with a sensation of heaviness in limbs?	1.52
Do you know that DVT may present with swelling in limbs?	1.51
**Dimension of risk factors**	
Do you know that older age will increase the risk of VTE?	
Do you know that staying in bed for ≥ 3 days or being sedentary will increase the risk of VTE?	
Do you know that obesity (BMI ≥ 28 kg/m^2^) will increase the risk of VTE?	
**Dimension of preventive measures**	
Do you know that exercise can prevent VTE?	1.93
Do you know that drinking enough water can prevent VTE?	1.81
Do you know that a low-fat diet can prevent VTE?	1.80
**The three lowest-scoring items in each dimension**	
**Dimension of basic knowledge**	
Do you know what is pulmonary embolism?	1.38
Do you know that malignant tumors may cause thrombus?	1.45
Do you know that breaking off a thrombus may cause pulmonary embolism?	1.48
**Dimension of clinical symptoms/signs**	
Do you know that post-thrombotic syndromes may present with pain, chronic ulcers, and pigmentation?	1.40
Do you know that pulmonary embolism may present with syncope?	1.42
Do you know pulmonary embolism may present with chest pain?	1.43
**Dimension of risk factors**	
Do you know that ≥ 3 times unexplained miscarriages or habitual miscarriages will increase the risk of VTE?	1.29
Do you know that oral contraceptives and hormone replacement therapy increase the risk of VTE?	1.34
Do you know that postmenopausal women have an increased risk of VTE?	1.35
**Dimension of preventive measures**	
Do you know that wearing graded compression stockings can prevent blood clots?	1.64
Do you know that VTE can be prevented with medication?	1.68
Do you know that prevention and early detection of VTE are important?	1.71

### Factors associated with VTE awareness level

In the univariate analysis (Table 1), all variables were associated with participants’ VTE awareness level (*p* < 0.05) and were subsequently included in the multiple linear stepwise regression analysis.

The results of multivariate analysis are presented in Table 3. Patients with higher education levels (Junior/senior high school: β = 0.09, *p* = 0.014; College graduated or above: β = 0.28, *p* < 0.001) and patients with a personal history of VTE (β = 0.20, *p* < 0.001), family history of VTE (β = 0.10, *p* = 0.014), history of chemotherapy (β = 0.10, *p* < 0.001), surgery (β = 0.11, *p* < 0.001), had higher levels of awareness of VTE. Patients admitted to hospitals in Western China exhibited a lower awareness level of VTE (β = -0.19, *p* < 0.001).

**Table 3:** Multiple linear stepwise regression analysis of participants’ VTE awareness level

Independent variable	B	S.E.	β	t	P	95%CI LB	95%CI UB	VIF
Constant	1.64	0.07		22.08	< 0.001	1.49	1.79	
Education level (reference = primary school or below)								
Junior/senior high school	0.09	0.04	0.08	2.46	0.014	0.02	0.16	0.39
College graduate or above	0.28	0.04	0.26	7.63	< 0.001	0.21	0.35	0.36
Personal history of VTE	0.20	0.06	0.07	3.47	< 0.001	0.09	0.31	0.93
Family history of VTE	0.10	0.04	0.05	2.45	0.014	0.02	0.19	0.94
Chemotherapy history	0.10	0.03	0.09	3.35	< 0.001	0.04	0.16	0.58
Surgical history	0.11	0.03	0.09	4.18	< 0.001	0.06	0.16	0.92
Location (reference = Eastern China)								
Western China	-0.19	0.03	-0.14	-6.10	< 0.001	-0.25	-0.13	0.78

F = 26.665, *p* < 0.001, R^2^ = 0.19, adjusted R^2^ = 0.18. Only variables with *p* < 0.05 are presented in the table

## Discussion

This study aimed to assess the awareness level of VTE in breast cancer surgery patients and identify factors that influenced their awareness level of VTE. The findings revealed that (1) the term awareness rates of DVT and PE were low compared with other diseases; (2) medical staff and online social applications were the main sources for participants to learn about VTE; (3) the participants’ overall awareness of VTE related knowledge is at an average level, but the awareness level regarding clinical symptoms/signs and risk factors of VTE is insufficient. (4) several factors associated with participants’ overall VTE awareness level, including education level, personal history of VTE, chemotherapy history, surgical history, and the region where the hospital is located.

In the present survey covering several common cardiovascular diseases, the terminology awareness rates for DVT and PE were the lowest. This is consistent with the findings from Wendelboe et al.‘s [28] 2014 global survey of 7,233 ordinary people, though the terminology awareness rates for DVT and PE in this study (42.5% for DVT and 26.1% for PE) are lower than those reported by Wendelboe et al. (44% for DVT and 54% for PE, respectively). Lavall et al. [30] conducted a survey on 325 members of the general public and the results showed an awareness rate of 42% for both DVT and PE. Aggarwal et al. ‘s [29] survey of 500 cancer patients in the United States indicated an awareness rate of 24% for DVT and 15% for PE. These surveys demonstrated that both cancer patients, who are at a higher risk for VTE, and the general population have low awareness levels of VTE, though increasing measures to improve the public’s awareness of VTE have been implemented at national and international levels. This gap necessitates targeted awareness campaigns and educational interventions, especially for high-risk groups like cancer surgical patients.

In comparison to breast cancer surgical patients’ awareness regarding other postoperative complications, we found limited studies focusing on this topic, only uncovering one study that investigated breast cancer surgical patients’ awareness of lymphedema [31]. This study surveyed 135 breast cancer patients and found that 70.4% of patients were unaware of lymphedema, which was higher than the proportion of patients who were unaware of VTE in this study. What is mentioned above indicates that there may be a general lack of awareness about postoperative complications among breast cancer surgical patients, and there is a deficiency in research exploring patients’ awareness of common postoperative complications. Thus, this study advocates future research to comprehensively investigate the awareness of postoperative complications among breast cancer patients. And, we recommend that medical professionals place greater emphasis on health education regarding postoperative complications to improve breast cancer patients’ awareness, aiming to enhance patients’ awareness, reduce the risk of complications, and assist patients in the early identification of such conditions.

A key finding from this survey is the reliance on healthcare professionals, predominantly physicians, and nurses, as the primary source of knowledge about VTE. This underscores the crucial role of medical professionals in patient education and highlights the need for enhanced educational initiatives, particularly for VTE high-risk groups such as cancer patients receiving surgery. With the rising popularity of social media, it has become an important way for the public to acquire health information alongside hospital medical staff, especially in the setting of community care or home care [32]. Plenty of studies have proved its effectiveness and convenience in patients education and patient education and engagement [33–35]. The Baddeley et al. [36] developed a patient health education video, called “Blood Clots, Cancer and You”, which served as a supplement to written and oral health education, and could be watched repeatedly as desired at wards and outpatient clinics. This video has to be proven to significantly shorten the time from VTE symptom onset to diagnosis, reflecting patients’ greater awareness of VTE. Therefore, utilizing crafted videos as an auxiliary tool for medical staff to implement health education may help improve patients’ awareness of VTE, and further dissemination of health education videos through social media may allow patients to continue to improve their awareness of VTE in out-of-hospital settings, tailored to their needs.

The survey results indicated that among the four investigated dimensions, the awareness scores for VTE’s clinical symptoms/signs of VTE (1.48 ± 0.60) and risk factors of VTE (1.50 ± 0.55) were the lowest. Therefore, healthcare professionals need to emphasize education about the clinical symptoms/signs and risk factors of VTE. Although the dimension for VTE preventive measures scored the highest, patients can better effectively understand and adhere to these preventive measures when they fully recognize the clinical symptoms/signs and risk factors of VTE, which will enhance their self-management ability for VTE. With the promotion of the Enhanced Recovery After Surgery (ERAS) medical model, the hospital stay for surgical patients is becoming shorter, which increases the likelihood of VTE occurrences post-discharge [37–39]. Furthermore, given that early symptoms of VTE are often atypical, the condition may be underdiagnosed or misdiagnosed after discharge, potentially developing into serious DVT or PE. Thus, enhancing patients’ knowledge of VTE’s clinical symptoms/signs would aid in the timely identification of early VTE symptoms post-discharge, avoiding missing the optimal window for treatment of VTE due to late detection.

The study also identified that patients with a higher level of education, a personal history of VTE, a history of chemotherapy, a history of surgery, and those receiving care in hospitals located in the more economically developed eastern or central regions of China, scored higher on the VTE awareness level. The association between lower educational levels and lower VTE awareness suggests a need for simplified and more accessible educational materials targeting this subgroup, thereby enhancing the effectiveness of health education.

## Conclusion

This study highlights a critical need for improved VTE awareness among breast cancer surgery patients, especially in the aspects of clinical symptoms/signs and risk factors of VTE. Health education programs are recommended especially tailored for patients with lower education levels, no history of VTE, or without prior surgery or chemotherapy, to improve their understanding of VTE.

## Limitations

This study has several limitations. First, despite being a multicenter cross-sectional survey, significant variation in sample size across centers may limit the representativeness of the findings. Second, we only investigated the awareness level of VTE in breast cancer patients, which also limits the generalizability of the research results. It is also important to consider a broader range of cancer types to understand the variability in VTE awareness across different patient populations. Future studies could aim for a more balanced sample distribution across centers or include a wider range of cancer types to enhance the generalizability and applicability of the research.

## Data Availability

The data used to support the findings of this study are available from the corresponding author upon reasonable request.

## Abbreviations

VTE: Venous thromboembolism
DVT: Deep vein thrombosis
PE: Pulmonary embolism
CVC: Central venous catheterization
